# Diagnostic value of FNA, ultrasound elastography and CEUS in thyroid carcinoma

**DOI:** 10.4314/ahs.v24i3.23

**Published:** 2024-09

**Authors:** Lei Zhao, Lin Zhao, Hui Chen, Rongfei Huang, Qian Wei, Jian Liu

**Affiliations:** 1 Department of Ultrasonic Medicine, Chengdu Medical College, Clinical Medical College and The First Affiliated Hospital of Chengdu Medical College, Chengdu, China; 2 Department of Pathology, Chengdu Medical College, Clinical Medical College and The First Affiliated Hospital of Chengdu Medical College, Chengdu, China

**Keywords:** Fine needle aspiration cytology, ultrasound elastography, contrast-enhanced ultrasound, thyroid cancer, diagnostic efficacy

## Abstract

**Background:**

To investigate the role of fine needle aspiration cytology (FNA), sonoelastography and contrast-enhanced ultrasound (CEUS) in the diagnosis of thyroid carcinoma.

**Methodology:**

A total of 150 patients with suspected thyroid cancer admitted to our hospital from January 2019 to December 2021 were selected and divided into group A, group B and group C according to random number table, with 50 cases in each group. FNA was used in group A, ultrasound elastography was used in group B, and CEUS was used in group C. Pathological diagnosis was performed in all patients, and the diagnostic sensitivity, specificity, and accuracy were compared among the three groups.

**Results:**

The diagnostic sensitivity, specificity and accuracy of FNA in group A were 90.91%, 70.59% and 84.00%, respectively; the diagnostic sensitivity, specificity and accuracy of ultrasound elastography in group B were 94.12%, 81.25% and 90.00%, respectively; the diagnostic sensitivity, specificity and accuracy of CEUS in group C were 88.57%, 73.33% and 84.00%, respectively; the three groups were compared (P > 0.05).

**Conclusion:**

All three diagnostic techniques had a relatively high sensitivity and accuracy in the diagnosis of thyroid carcinoma. Ultrasound elastography had the highest sensitivity and specificity among the three techniques, while FNA and CEUS had similar diagnostic performance.

## Introduction

Thyroid cancer mainly refers to malignant tumors originating from thyroid follicular epithelial cells or parafollicular epithelial cells. Thyroid cancer is the most common endocrine malignancy worldwide, accounting for about 1% of all new cancer cases each year. In the United States, thyroid cancer is the fifth most common cancer in women and the ninth most common in men. In 2022, it is estimated that there will be about 44,280 new cases of thyroid cancer in the United States. Thyroid cancer is more common in women than men, with a female to male ratio of about 3:1. The incidence of thyroid cancer has been increasing in recent years, largely due to the increased detection of small, asymptomatic nodules through the use of ultrasound and other imaging techniques. The mortality rate from thyroid cancer is relatively low, with a 5-year survival rate of about 98% for papillary and follicular thyroid carcinomas, the most common types of thyroid cancer. Most patients have no obvious symptoms in the early stage, and mostly present with painless cervical mass or nodules. With the progression of the disease, it may compress or invade adjacent organs or tissues, resulting in jugular venous distension, hoarseness, facial flushing, tachycardia and other manifestations. Some patients may also have cervical lymph node metastasis and distant organ metastasis, which will not only affect the physical and mental health of patients, but also cause distress to their daily life^1^. As the disease is insidious, it is usually advanced by the time you seek medical attention, so it is important to make an accurate diagnosis at an early stage of the disease. The study by Naykky Singh Ospina *et al.*^2^ scholars pointed out that in order to ensure that thyroid cancer patients can receive timely and correct diagnosis and treatment, it is necessary to improve the accuracy of thyroid cancer risk assessment. FNA is currently the most accurate and cost-effective diagnostic method and is more widely used in clinical practice^3^. With the continuous innovation of medical technology, ultrasound diagnosis, as a non-invasive technology, has been widely used in the clinical diagnosis and treatment process, of which ultrasound elastography can improve the accuracy of the diagnosis of thyroid nodules. CEUS can reflect the microperfusion of the tumor^4^,^5^.

FNA is currently the gold standard for the diagnosis of thyroid nodules. However, it has limitations in detecting small or indeterminate nodules. Ultrasound elastography and CEUS can provide additional information to supplement FNA and improve the accuracy of the diagnosis. Ultrasound elastography and CEUS are emerging diagnostic techniques that can provide real-time information about the mechanical and vascular properties of thyroid nodules. These techniques can help distinguish between benign and malignant nodules and provide a more accurate diagnosis. The diagnostic value of ultrasound elastography and CEUS in the diagnosis of thyroid carcinoma is still controversial, and further studies are needed to determine their effectiveness. Comparing these techniques to FNA can help determine their strengths and weaknesses in diagnosing thyroid carcinoma and guide the development of more accurate diagnostic tools. Accurate diagnosis of thyroid carcinoma is important for appropriate treatment and improved patient outcomes. By comparing FNA, ultrasound elastography, and CEUS, clinicians can choose the most appropriate diagnostic tool for each patient and provide more personalized and effective treatment. Considering this, this study analysed FNA, ultrasound elastography, and CEUS as diagnostic methods for thyroid cancer in order to further improve confidence in clinical diagnosis. .

## Patients and methods

### Study setting

The type of study was a prospective randomized controlled study. This study has been approved by the hospital ethics committee. A total of 150 patients with suspected thyroid cancer admitted from January 2019 to December 2021 were selected as the study subjects and divided into groups according to the random number table (Clinical registration number of this study: ChiCTR1800020447). There were 23 male patients and 27 female patients in group A (n=50) while in group B (n=50), there were 22 male patients and 28 female patients; Group C (n=50) had 24 male patients and 26 female patients. Patients confirmed to have met the diagnostic criteria for thyroid cancer in the Expert Consensus on the Clinical Application of Serum Markers for Thyroid Cancer 6. Inclusion criteria were as follows: (i) clinical information was relatively complete, and the patients' age ≥18 years old; (ii) basic communication was possible; (iii) patients all gave informed consent to the study. Exclusion criteria were as follows: (i) patients with psychiatric disorders, severe impairment of liver and kidney function, or during pregnancy or lactation; (ii) patients with contraindications to the diagnostic tools used in this study; (iii) patients with poor compliance with medical treatment. This study has been approved by the hospital ethics committee.

## Method

**Group A patients underwent FNA examination:** The examination was performed when the patients' vital signs were stable. The patient was assisted to lie in a supine position with the head slightly tilted back to fully expose the neck, routinely disinfected and towelled, and local anesthesia was administered with lidocaine hydrochloride injection (manufacturer: Shanghai Chaohui Pharmaceutical Co., Ltd.; approval number: State Drug Quantifier H31021071; specification: 20 ml:0.4 g). 20182231047; specification: DW-T3) to determine the location of the lesion, the puncture point and the angle of needle insertion. After puncturing the nodule with a non-negative pressure puncture needle, the needle tract is changed and the nodule is repeatedly extracted 2-3 times, and then a smear is made and sent for examination. The diagnostic criteria for FNA are as follows: a smear showing a follicular tumour or a suspicious follicular tumour, or a suspicious malignant tumour, will lead to a diagnosis of thyroid cancer.

**Group B underwent ultrasound elastography:** In group B, ultrasound elastography was performed: a colour Doppler ultrasound diagnostic instrument was used to detect the nodule, and the frequency of the probe was set at 6-13MHz; the patient was assisted to lie in a supine position with the head slightly tilted back to fully expose the neck; firstly, a routine scan was performed to locate the nodule and clarify the basic condition of the nodule; secondly, the elastography mode was activated to observe the blood flow in the nodule area under colour Doppler flow imaging (CDFI), looking for The largest section is fixed by looking for the section with the most blood flow; the probe is then held perpendicular to the fixed nodal area, making small movements and applying pressure in a smooth manner to perform the imaging, which is colour coded to indicate the size of the elasticity. The diagnostic criteria for ultrasound elastography are as follows: elastography blue is scored as 4; elastography blue and green, but mostly blue is scored as 3; elastography blue and green, but mostly green is scored as 2; elastography blue, green and red is scored as 1; elastography blue, green and red, but mostly red is scored as 0. Generally, the higher the score, the harder the nodule and the higher the risk of malignancy, with a score of 3-4 being sufficient for a diagnosis of thyroid cancer.

**In group C, CEUS was performed:** a colour Doppler ultrasound diagnostic instrument was used to detect the nodule, with the probe frequency set at 5-12 MHz. The patient was assisted to lie in a supine position with the head slightly tilted back to fully expose the neck; firstly, a routine ultrasound examination was performed to determine the size, morphology and echogenicity of the nodule, followed by mixing the contrast agent (manufacturer: Bracco Suisse SA; approval number: H20120527; specification: 59mg) with 0.9% sodium chloride injection (manufacturer: Sichuan Keren Pharmaceutical Co. The contrast medium (manufacturer: Bracco Suisse SA; approval number: H20120527; specification: 59mg) was mixed with 0.9% sodium chloride injection (manufacturer: Sichuan Keren Pharmaceutical Co., Ltd.; approval number: Guopharmachem H51021158; specification: 500ml:4.5g), shaken vigorously until the contrast medium was completely dispersed, then 2ml was injected rapidly into the elbow vein, followed by 5ml of saline at random. Store the entire procedure. If the patient has multiple foci, CEUS may be repeated 15 min later. The diagnostic criteria for CEUS are as follows: early arterial inhomogeneous hypoenhancement is diagnostic of thyroid cancer.

### Observative indication

Referring to the pathological results, the diagnostic results of the three groups were observed and the diagnostic efficacy of the three groups was compared: 4. Sensitivity = True Positives / (True Positives + False Negatives); Specificity = True Negatives / (True Negatives + False Positives); Accuracy = (True Positives + True Negatives) / (True Positives + False Positives + True Negatives + False Negatives)

### Statistical analysis

SPSS 23.0 was applied for statistical analysis. Independent sample t-test was used for comparison between groups for measurement data obeying normal distribution, and independent sample t-test was used for comparison within groups, all expressed as (x̅±s ). Count data were tested by χ^2^ and expressed as rate (%), P<0.05 indicates statistical difference.

## Results

### Baseline data

The Baseline data of the three groups were compared. There was no different among the three groups (P > 0.05), as shown in Table 1.

**Table 1:** The baseline data of the study

Groups	n	Gender (male, %)	Age(years)	disease duration(years)
Group A	50	23(46)	44.23±6.74	1.96±0.51
Group B	50	22(44)	44.57± 6.58	2.03±0.69
Group C	50	24(48)	45.02±6.76	2.07±0.61
F/χ^2^		0.161	0.175	0.420
P		0.923	0.839	0.658

### Diagnostic results

The diagnostic results of FNA in group A showed that there were 30 true positive cases and 12 true negative cases; the diagnostic results of ultrasound elastography in group B showed that there were 32 true positive cases and 13 true negative cases; the diagnostic results of CEUS in group C showed that there were 31 true positive cases and 11 true negative cases. See Table 2:

**Table 2:** Comparison of diagnostic results of three examination methods

Pathological diagnosis results	Group A			Group B			Group C		

	Positive	Negative	Total	Positive	Negative	Total	Positive	Negative	Total
Positive	30	5	35	32	3	35	31	4	35
Negative	3	12	15	2	13	15	4	11	15
Total	33	17	50	34	16	50	35	15	50

### Diagnostic efficacy

The diagnostic sensitivity, specificity and accuracy of FNA in group A were 90.91%, 70.59% and 84.00%, respectively; the diagnostic sensitivity, specificity and accuracy of ultrasound elastography in group B were 94.12%, 81.25% and 90.00%, respectively; the diagnostic sensitivity, specificity and accuracy of CEUS in group C were 88.57%, 73.33% and 84.00%, respectively; the three groups were compared (P > 0.05) (Table 3).

**Table 3:** Comparison of diagnostic efficacy of three examination methods (n, %)

Group	N	Sensitivity	Specificity	Accuracy
Group A	50	30/33 (90.91)	12/17 (70.59)	42/50 (84.00)
Group B	50	32/34 (94.12)	13/16 (81.25)	45/50 (90.00)
Group C	50	31/35 (88.57)	11/15 (73.33)	42/50 (84.00)
X^2^		0.043	0.219	0.796
P		0.834	0.640	0.372

### Disease types

The results of pathological diagnosis showed 30 cases of papillary thyroid carcinoma and 5 cases of thyroid follicular carcinoma; the results of FNA diagnosis in group A showed 27 cases of papillary thyroid carcinoma and 3 cases of thyroid follicular carcinoma; the results of ultrasound elastography diagnosis in group B showed 28 cases of papillary thyroid carcinoma and 4 cases of thyroid follicular carcinoma; the results of CEUS diagnosis in group C showed 27 cases of papillary thyroid carcinoma and 4 cases of thyroid follicular carcinoma.

## Discussion

The exact etiology of thyroid cancer cannot be fully determined, and it is clinically believed that its basic etiology mainly lies in the following aspects: oncogene, the occurrence and development of thyroid cancer is correlated with the overexpression, mutation or deletion of oncogene sequence; polypeptide growth factors, such as thyroid-stimulating hormone and insulin-like growth factor, can not only act on the growth and differentiation of normal thyroid follicular cells, but also participate in tumor proliferation and metastasis with oncogenes; ionizing radiation is one of the clear pathogenic factors, and the occurrence of thyroid cancer is related to the history of radiation exposure and exposure; gender, the incidence of women is generally high, which may be related to thyroid dysfunction caused by unique stages such as pregnancy, abortion and perimenopausal period in women and cause the body's immunity; genetic factors, some patients with thyroid cancer have familial predisposition, such as familial adenoma polyps and Gardner syndrome will increase the incidence of the disease. In addition, irregular diet, nutritional imbalance, smoking, alcohol abuse, and more negative emotions can also contribute to the development of thyroid cancer^7^,^8^. Studies 9 have shown that early diagnosis and treatment of thyroid cancer helps to improve the prognosis. Biopsy in pathological examination is the “gold standard” for the diagnosis of thyroid cancer, but needle biopsy may cause cancer cell spread and therefore cannot be widely used. Ultrasonography in imaging examination is the most commonly used and preferred method for the thyroid gland and has unique advantages for the early diagnosis, reasonable assessment, accurate staging and timely treatment of thyroid cancer.

FNA belongs to one of the pathological examinations, which uses a non-negative pressure puncture needle to puncture the nodule, places a small number of cells on a slide and makes a smear for cytological examination, so as to diagnose the disease. Its advantages are: (1) The diagnostic accuracy is high: it performs puncture under the guidance of color Doppler ultrasonic diagnostic apparatus, which helps to puncture to the effective area, and then improves the accuracy of puncture and diagnosis^10^. (2) Easy operation: the nature of the nodule can be rapidly judged based on the characteristics of the cells, and a pathological examination report can be produced in 0.5 h. However, the diagnostic results depend on the puncture sampling method and the experience of reading to identify cells, so it has a certain rate of misdiagnosis and missed diagnosis.

Ultrasound elastography mainly uses the radiofrequency signal after compression of the examined site to determine the nature, and comprehensively analyses the nodule hardness for diagnosis by observing the color change and distribution of the examined site. Its advantages are: (1) It can better detect microlesions: mainly in papillary carcinoma of thyroid cancer, the stromal component is small, it can form a more obvious elastic strain difference with the surrounding normal tissues, and then improve the detection rate. (2), which can accurately differentiate the nature of thyroid nodules: there are a large number of blood vessels and fibers distributed in the malignant nodules, and with the deepening of the degree of calcification, the elastic hardness of the nodules will continue to increase; the glial follicular lumen of benign nodules, which is soft in consistency, is lower than that of malignant nodules even with partial calcification^11^. However, elastography also has certain limitations, and its diagnostic results are related to factors such as basic hardness, nodule size, internal calcification, liquefaction necrosis, image cross overlap phenomenon, and operator's technique and experience of the examined site, so the influence of the above factors should be fully considered to avoid and reduce missed diagnosis and misdiagnosis as much as possible.

CEUS is mainly used to enhance the blood flow scattering signal by injecting contrast agent into the peripheral vein and using the acoustic impedance difference between contrast agent and soft tissue, so as to dynamically observe the microvascular perfusion in real time to detect the diseased tissue. Its advantages are: (1) It can be used to evaluate organ or tissue function: the contrast agent used is a pure blood cell contrast agent, which can display microvascular perfusion and regression in real time, so as to accurately present the vascularity. (2) Differential diagnosis of nodular nature: After injection of contrast agent, benign nodules will present synchronous homogeneous enhancement, ring enhancement, or no enhancement with the surrounding normal tissue; malignant nodules may show heterogeneous enhancement or hypoenhancement^12^. However, the operation process of CEUS is cumbersome and the requirements for the operator are relatively high, so it will also produce partial missed diagnosis and misdiagnosis.

This study has the following limitations, like the small sample size of the study, it might not provide a comprehensive picture of the target population, the interpretation of the ultrasound images and FNA results may be subjective, and the reliability of the findings may depend on the experience and expertise of the radiologists and pathologists involved in the study.

## Conclusion

All three diagnostic techniques had a relatively high sensitivity and accuracy in the diagnosis of thyroid carcinoma. Ultrasound elastography had the highest sensitivity and specificity among the three techniques, while FNA and CEUS had similar diagnostic performance. Overall, the study provided valuable insights into the diagnostic value of FNA, ultrasound elastography, and CEUS in the diagnosis of thyroid carcinoma. The findings suggested that these techniques can be useful in the diagnostic workup of patients with thyroid nodules, and that ultrasound elastography in particular might be a promising tool for improving diagnostic accuracy.

## Data Availability

The datasets used and analysed during the current study are available from the corresponding author on reasonable request.
